# Impact of General and Oral Complications of Diabetes Mellitus Type I on Lebanese Children’s Quality of Life

**DOI:** 10.5005/jp-journals-10005-1481

**Published:** 2017-02-01

**Authors:** Balsam Noueiri, Nahla Nassif, Abbas Ollek

**Affiliations:** 1 Associate Professor, Department of Pediatric Dentistry, Lebanese University Beirut, Lebanon; 2 Assistant Professor, Department of Pediatric Dentistry, School of Dentistry, Lebanese University, Beirut, Lebanon; 3 Assistant Professor, Department of Life Science, Lebanese University, Beirut Lebanon

**Keywords:** Children, Chronic diseases, Diabetic, Oral complications, Oral health, Quality of life.

## Abstract

**Introduction:**

Diabetes mellitus type I (DM1) has been increasing at an alarming rate worldwide. Children suffering from this chronic disease are subject to a high risk of systemic and oral complications, due to their young age and the lack of awareness of the relation between diabetes and oral health.

**Objective:**

The aim of this study is to evaluate the impact of oral and general complications of DM1 on the Lebanese children’s quality of life. The goal was to assess the child’s behavioral issues on the one hand and the oral issues on the other.

**Materials and methods:**

About 37 diabetic Lebanese children aged between 6 and 12 years, recruited from the Chronic Care Center (CCC), answered two questionnaires, one related to the disease and the second related to the oral complications.

**Results:**

A majority of the participants (81.1%) are aware of their disease, 73% know the importance of their treatment and 54.1% are able to control their glycemia; 45.9% are not annoyed with constantly carrying a monitor and 67.5% are bothered by their restricted diet. Only 5.4% of children isolate themselves.

**Concerning the oral complications:**

About 83.8% of the children do not suffer from oral ulcers, 56.8% are caries-free, and 64.9% have completed their dental treatment; 89.2% do not complain while eating and 94.6% are not able to brush their teeth properly.

**Conclusion:**

Diabetic patients are found to have good knowledge of the disease and its systemic complications but a little on their increased risk for oral diseases. In order to ensure a good quality of life for the diabetic children and their families, optimal control of diabetes, appropriate oral hygiene, and regular visits to the dentist must be respected.

**How to cite this article:** Noueiri B, Nassif N, Ollek A. Impact of General and Oral Complications of Diabetes Mellitus Type I on Lebanese Children’s Quality of Life. Int J Clin Pediatr Dent 2018;11(1):40-45.

## INTRODUCTION

Diabetes mellitus is a chronic illness that requires continuing medical care and ongoing patient self-management, education, and support to prevent acute complications and to reduce the risk of long-term ones. Diabetes care is complex and requires multifactorial risk-reduction strategies beyond glycemic control.^1^ The spread of this disease is rising in a dramatic way, especially in developing countries, giving the term “diabetic epidemic” an actual credibility.^2^ In fact, epidemiologic studies estimate an increasing prevalence of disease from 4% in 1995 to 5.4% in 2025.^3^

Zhang et al^4^ predicted that at least 366 million people around the world will have DM by the year 2030.

Due to its chronic nature and multisystemic involvement, diabetes may affect the health-related quality of life (HRQoL) of the patient and can exert a negative impact on it.^5,6^ In fact, DM is associated with long-term damage of various organs, including the eyes, kidneys, nerves, heart, and blood vessels.^7^

Concerning the oral complications, children with DM1 represent a group in which factors related to caries disease and gingivitis take part in their daily life.^8,9^

Findings from a previous study indicate that children with diabetes had significantly more dental plaque and gingival inflammation than nondiabetic children.^10^ In addition, they have more likely mucosal disorders.^11^

Decreased immunity, diminution of salivary flow, high amount of food intake per day, presence of plaque, and poor hygiene are among these oral affections. It may have an impact on the patient’s quality of life. Pain and masticatory difficulties can provide appetite loss, weight loss, insomnia, behavioral changes, and may lead to decreased school attendance.^12,13^ Moreover, it can provide psychological problems (self-esteem) especially in children and adolescents, such as more oppositional problems, aggression, and rule-breaking behavior than controls.^14^ The children reported more externalizing disorders and the presence of family conflicts.^15,16^

Despite its relatively recent emergence over the past few decades, oral health-related quality of life (OHRQoL) is considered now as an integral part of general health and well-being and is recognized by the World Health Organization as an important segment of the Global Oral Health Program.^6^

However, few studies have investigated the association between HRQoL and OHRQoL.^17^

The aim of this study is to evaluate the impact of oral and general complications of DM1 on the Lebanese children’s quality of life. The objective of the study is to assess the child’s behavioral issues, such as the awareness toward the disease, the well-being, the social life, the psychological condition, and the schooling problems and to evaluate the oral issues (i.e., ulcers, cavities, plaque, and gingivitis).

## MATERIALS AND METHODS

A cross-sectional approach was utilized to conduct this study. Our population of interest was 37 Lebanese diabetic children aged between 6 and 12 years, treated by specialists in the CCC (CCC is a Lebanese private nonlucrative institution with a multidisciplinary medical team for preventing and monitoring certain chronic childhood diseases including DM1) since at least 18 months. With the help of one researcher, the parents answered two questionnaires: the first one (consisting of 14 questions) was related to the impact of the disease and its treatment on the children’s daily activities and their well-being. The second (consisting of 11 questions) was about the impact of the oral complications of diabetes on the child’s quality of life.

A consent form with a written approval was signed by the parents for the inclusion of their children in the study.

The answers were scored on a three-point scale: 0 = never or not at all, 1 = moderately, and 2 = excessively.

The answers were collected and then statistically analyzed using Statistical Package for the Social Sciences (version 18.0). The chi-square test was used to determine the level of association between variables. The level of significance was set as p < 0.05.

**Table 1:** **Table 1:** Impact of DM1 and its treatment on the child’s daily activities and well-being

Q_1_		*Values*		*Proportions in %*		*p-value*	
Is the child aware of his/her		0		2.7		0.000	
condition?		1		16.2			
		2		81.1			
Is the child aware of the		0		5.4		0.000	
importance of his/her		1		21.6			
treatment?		2		73			
Is the child able to control his/		0		8.1		0.000	
her blood sugar levels on his/		1		37.8			
her own?		2		54.1			
Is it easy for the child to check		0		5.4		0.002	
his/her glycemia on his/her		1		10.8			
own?		2		83.8			
Does the child take insulin		0		13.5		0.000	
shots without complaining?		1		18.9			
		2		67.6			
Does the child accept easily to		0		24.3		0.245	
carry his/her monitor?		1		29.7			
		2		45.9			
Is the child socially confident		0		29.7		0.649	
with his/her condition?		1		40.5			
		2		29.7			
Does the child practice normal		0		13.5		0.000	
physical activities?		1		18.9			
		2		67.6			
Does the child attend regularly		0		8.1		0.000	
social events?		1		21.6			
		2		70.3			
Is the child popular and		0		5.4		0.000	
friendly?		1		27			
		2		67.6			
Does the child manage to		0		48.6		0.139	
control his/her temper?		1		27			
		2		24.3			
Does the child accept his/her		0		32.4		0.973	
restricted diet?		1		35.1			
		2		32.4			
Is the child attending school		0		2.7		0.000	
regularly?		1		43.2			
		2		54.1			
Is the child’s vigilance normal?		0		18.9		0.01	
		1		24.3			
		2		56.8			

## RESULTS

Table 1 shows that 81.1% of the children are totally aware of their disease, 73% know very well the importance of their treatment, 54.1% are able to control their glycemia by their own, and 83.8% are able to check their blood sugar level by themselves.

The answers related to the well-being were as follows: 67.6% of the children take insulin shots without complaining, 45.9% are not annoyed by constantly carrying a monitor, and 29.7% do not feel different from their friends.

Diabetes deprives 13.5% of the children from practicing sports and 8.1% from attending social events; 67.5% are annoyed by their restricted diet, 24.3% find that their emotional state is not affected by their condition, and 5.4% of children often isolate themselves; 54.1% attend school regularly and 56.8% of the children receive a regular education.

**Table 2:** **Table 2:** Oral complications due to DM1 on the child’s quality of life

Q_2_		*Values*		*Proportions in %*		*p-value*	
Are the child’s oral mucosa		0		0		0.000	
and gum exempt from ulcers		1		16.2			
and bleeding?		2		83.8			
Are the child’s teeth exempt		0		56.8		0.001	
from decay?		1		35.1			
		2		8.1			
Does the child have treated		0		35.1		0.469	
teeth?		1		40.5			
		2		24.4			
Are the child’s teeth always		0		37.8		0.509	
clean?		1		37.8			
		2		24.3			
Does the child feel comfortable		0		2.7		0.000	
while eating?		1		8.1			
		2		89.2			
Does the child cooperate with		0		21.6		0.118	
his/her dentist?		1		48.6			
		2		29.7			
Does the child know how to		0		94.6		0.000	
brush properly his/her teeth by		1		5.4			
himself/herself?		2		0			
Is the child sleeping properly		0		2.7		0.000	
at night?		1		2.7			
		2		94.6			
Does the child know that oral		0		81.1		0.000	
complications affect negatively		1		10.8			
his/her systemic condition?		2		8.1			
Is the child attending school		0		0		0.000	
regularly?		1		10.8			
		2		89.2			
Do you take your child for		0		45.9		0.014	
dental checkups?		1		10.8			
		2		43.2			

In Table 2, 83.8% of the children do not suffer from oral ulcers, 8.1% are caries free, and 64.9% have treated teeth; 89.2% do not complain from oral pain while eating and 94.6% are not able to brush their teeth properly without the help of a parent. Concerning the relationship between the child and the dentist, 78.3% of the children cooperate during dental treatments; 94.6% of children sleep well at night and 89.2% do not skip school at all; 81.1% of the children have very poor knowledge of oral health and are not aware of the impact of oral complications on their systemic condition. Finally, 45.9% are not followed regularly by a dentist.

## DISCUSSION

Many studies on the quality of life among diabetic patients have been conducted but few have evaluated OHRQoL.^3^ Less is known about the association between HRQoL and OHRQoL among patients with specific diseases.^17^

In the present study, we evaluate both impacts of DM1 and its oral complications on the Lebanese children’s quality of life.

In Table 1, concerning the awareness of the children about their general condition, 81.1% of them are totally conscious about their chronic disease and 73% understand the importance of their treatment (p 0.0000). This knowledge is attributed to the education received by the medical staff in the CCC. Additionally, 54.1% of the children are able to control their blood sugar levels on their own (p 0.002). The latter percentage reflects a limitation of self-management of the fluctuation of blood sugar levels by the young patients (6-12 years). In fact, near-normal glucose control is more difficult to achieve in child patients *vs* adult patients with DM1.^18^

Taheri^19^ mentioned that diabetic patients receive a limited education on oral health manifestations, special consideration of dental management, and control of blood sugar.

When it comes to insulin injections, 13.5% of the children present a serious fear while 86% accept it easily (p 0.000). Several studies showed that fear and anxiety among patients of pediatric age were generally attributed to the needle.^20-22^

These results can be explained by the fact that diabetic children have their injections on a daily basis since at least 18 months of treatment and got used to it.

The percentage of children having poor self-confidence (29.7%) and the percentage of those presenting a serious issue in carrying the monitor in public (24.3%) reflect a social discomfort. Though these results are not statistically significant, they could explain the society’s perception toward chronic diseases. Several testimonies are found in the literature.^23^

Rao et al^24^ revealed that DM affects people not only physically but also emotionally and psychosocially.

Considering the physical health of diabetic children, 67.6% practice physical activities regularly. The remaining 32.4% present a high fatigability due to their condition and cannot commit to any physical activity.

Cavini et al^23^ reported the behavioral, psychological, and social changes in diabetic patients, as well as their noncommitment to physical activities.

Concerning the social interaction, 70.3% of diabetic children attend social events and 67.6% are popular and friendly. Those findings point out to a relatively positive quality of life primarily due to the familial support and secondarily to the immediate entourage.

Dannan et al^25^ mentioned that anxiety and avoiding interactions with the society are some of the complications of DM1. The present study shows that a low percentage of children present antisocial behavior; 67.5% of diabetic children face difficulties in applying an appropriate diet. This fact is not surprising knowing that children, in general, prefer sweets and junk food. Indeed, the greatest difficulty reported by diabetic patient is their strict diet.^23,26^

About 27% of the children sampled can barely manage their temper while 48.6% are not able to control it at all. These results can be explained, partly because of the biological changes beyond their control and partly because, in this period of developmental transition, psychosocial factors can militate against young people upholding their lifestyle and medical regimens.^27^ For decades, authors found peculiar and unintelligible behavior in young diabetic patients.^28^ The pressures and changes of normal adolescent development can conflict with the self-awareness and orderliness needed to manage living with a chronic disease. These tensions create a platform for significant personal and familial stress and could even lead to mental illness.^27,29^

Concerning the child’s school attendance, only 2.7% face major problems in following their classes regularly and 43.2% present some absenteeism. Glaab et al^30^ found that children with DM1 miss more school than their non-DM siblings and peers.^31^

About 18.9% of children present a serious problem of concentration. Patients with DM1 have impaired sustained attention, which was associated with diabetes *per* se.^31^ Rustadet al^32^ revealed that patients with DM1 suffer neurocognitive deficits of executive functioning and working memory, which is associated with poorer glycemic control.^33^

In Table 2, we noticed that children with DM1 do not have serious periodontal problems; 83.8% of the children have healthy oral mucosa and gum, while 16.2% answered that there is a moderate gingivitis, which could be due to insufficient oral hygiene or an improper teeth brushing.

Findings from previous studies indicate that peri-odontal destruction is higher in children and adolescents. Moreover, children with diabetes had significantly more dental plaque and gingival inflammation than nondia-betic children.^10,34,35^

Only 8.1% of the children are caries-free and only 24.4% completed their dental treatment. Lamster^9^ insists on the role of nonoral health care providers who treat patients with diabetes: they must be aware of manifestations of oral diseases and inform patients that ideal oral health is part of comprehensive management of diabetes. All health care workers need to be aware of the bidirectional relationship between diabetes and oral health.^35^

About 89.2% of the children do not complain about dental pain while eating.

Considering the child’s cooperation with the dentist, the results were not statistically significant (p 0.118). Almost 100% of the children are not able to brush their teeth properly and 75.6% of the children presented with plaque on their teeth, which indicates that parents are not assisting their kids during teeth brushing.

Only 2.7% of the children have sleep deprivation due to dental or oral pain, while the majority (94.6%) benefit from a full night’s sleep. Bot et al^36^ reported that sleeping difficulties (p 0.004) are significantly related to higher glycated hemoglobin. We can conclude that sleep deprivation is related to DM itself.

The answers on the understanding of the relationship between oral health and diabetes are insufficient. Indeed, 91.9% ignore the impact of oral complications resulting from diabetes on the quality of life.

According to Sahril^35^ only 35.5% of patients perceive there was an association/relationship between diabetes and oral health. The authors also highlighted the lack of awareness and understanding of the bidirectional relationship between diabetes and oral health.^16,37^

Concerning education, the oral and dental issues barely disturbed the children’s attendance. About 10.8% reported some absenteeism. The percentage of absenteeism related to the diabetes itself was higher (45.9%; Table 1) than the absenteeism due to oral complication (10.8%; Table 2). This difference could be explained by the mandatory regular visits to the CCC to control the disease up close. However, the dental checkups are not so regular (56.7%), a point raised by many authors.^35,38,39^

## CONCLUSION

This study represents the first evaluation of HRQoL and OHRQoL in young Lebanese DM1 patients. The results were encouraging, especially concerning the awareness of the children about the disease and its systemic complications. The majority of them understand the importance of the treatment but lack knowledge about the impact of the disease on oral health.

The bidirectional relationship between diabetes and oral complications is misestimated by the children and their parents. Optimal control of diabetes, appropriate oral hygiene, and regular visits to the dentist have an important role in prevention of the oral complications of diabetes.

Health care providers must educate DM1 patients and their parents to enhance their understanding of the relationship between oral health and general health for the child’s well-being.

Oral prophylaxis can not only reduce the amount of oral problems but goes beyond it to improve glycemic control and general health.
